# New Trends in the Quality Control of Enantiomeric Drugs: Quality by Design-Compliant Development of Chiral Capillary Electrophoresis Methods

**DOI:** 10.3390/molecules27207058

**Published:** 2022-10-19

**Authors:** Serena Orlandini, Gabriel Hancu, Zoltán-István Szabó, Adriana Modroiu, Lajos-Attila Papp, Roberto Gotti, Sandra Furlanetto

**Affiliations:** 1 Department of Chemistry “U. Schiff”, University of Florence, 50019 Florence, Italy; 2 Department of Pharmaceutical and Therapeutic Chemistry, Faculty of Pharmacy, University of Medicine, Pharmacy, Science and Technology “George Emil Palade” of Târgu Mureș, 540139 Târgu Mureș, Romania; 3 Department of Pharmaceutical Industry and Management, Faculty of Pharmacy, University of Medicine, Pharmacy, Science and Technology “George Emil Palade” of Târgu Mureș, 540139 Târgu Mureș, Romania; 4 Department of Pharmacy and Biotechnology, University of Bologna, 40126 Bologna, Italy

**Keywords:** chiral separation, capillary electrophoresis, enantiomeric purity, experimental design, Analytical Quality by Design, ICH guidelines

## Abstract

Capillary electrophoresis (CE) is a potent method for analyzing chiral substances and is commonly used in the enantioseparation and chiral purity control of pharmaceuticals from different matrices. The adoption of Quality by Design (QbD) concepts in analytical method development, optimization and validation is a widespread trend observed in various analytical approaches including chiral CE. The application of Analytical QbD (AQbD) leads to the development of analytical methods based on sound science combined with risk management, and to a well understood process clarifying the influence of method parameters on the analytical output. The Design of Experiments (DoE) method employing chemometric tools is an essential part of QbD-based method development, allowing for the simultaneous evaluation of experimental parameters as well as their interaction. In 2022 the International Council for Harmonization (ICH) released two draft guidelines (ICH Q14 and ICH Q2(R2)) that are intended to encourage more robust analytical procedures. The ICH Q14 guideline intends to harmonize the scientific approaches for analytical procedures’ development, while the Q2(R2) document covers the validation principles for the use of analytical procedures including the recent applications that require multivariate statistical analyses. The aim of this review is to provide an overview of the new prospects for chiral CE method development applied for the enantiomeric purity control of pharmaceuticals using AQbD principles. The review also provides an overview of recent research (2012–2022) on the applicability of CE methods in chiral drug impurity profiling.

## 1. Introduction

Chirality is a property of asymmetry that governs our world; it is a property of an object or a molecule whose mirror image cannot be superimposed on itself, just like the right and the left hands of a human. A chiral molecule is a type of molecule that is not superposable with its mirror image. Most pharmaceuticals contain in their structure a center of chirality, leading to the existence of enantiomers. Usually, the asymmetric atom is a carbon with four different substituents; however, in some rare cases sulfur or phosphorus can also be chiral centers [1,2].

It is estimated that approximately 50% of the drugs currently used in therapy are chiral; however, only 25–30% of them are used in therapy as optically pure enantiomers, the rest being used as racemates. It is established that the pharmacological activity of a racemate is in many cases restricted to one of the enantiomers, called eutomer, while the other, called distomer, can be “inactive” or less active, and sometimes has a different pharmacological profile or can be responsible for the side effects that appear in the case of racemate administration. Enantiomers have similar physicochemical properties in an achiral environment and differ only through an optical one (rotation of polarized light); however, when introduced in a chiral environment such as the human body they can exhibit totally different pharmacokinetic (absorption, distribution, metabolism, excretion) and pharmacodynamic (quantitative or qualitative differences in pharmacologic or toxicologic effects) profiles generating different therapeutic responses [1,2,3].

The turning point in the history of chiral drugs was 27 May 1992 when the U.S. Food and Drug Administration (FDA) released a policy statement on the development of chiral drugs [4]. This statement changed everything for the scientist developing and validating analytical protocols for chiral medicinal compounds and products. According to FDA stipulations, the individual pharmacokinetic profile, pharmacological activity, and toxicity of each enantiomer should be studied before the introduction of a chiral drug in therapy. It is also crucial to establish limits for all isomeric components, impurities, and contaminants and to determine concentrations of each enantiomer [5]. Currently, regulatory bodies demand that chiral drug compounds undergo rigorous analytical characterization, including a thorough recording of the distinct pharmacological and pharmacokinetic properties of the individual enantiomers as well as their combination [5,6].

The market of new chiral drugs either creates de novo enantiomerically pure compounds or uses the “chiral switch” technique from existing commercially available racemates [7]. Although the FDA did not require specifically the development of single enantiomers (racemates may be suitable in some situations), pharmaceutical companies now develop, almost exclusively, single enantiomer medications when working with chemicals that include chiral centers [5,7]. The paradigm of optically pure enantiomers has shifted as the development of large-scale chiral separation techniques and asymmetric syntheses have emerged and enantiopure compounds have become more readily available. Pure enantiomer development involves questions about suitable pharmacologic and toxicologic assessment, synthesis and impurity control, accurate characterization of metabolism and distribution, and appropriate clinical evaluation [6,7].

**Figure 1:**
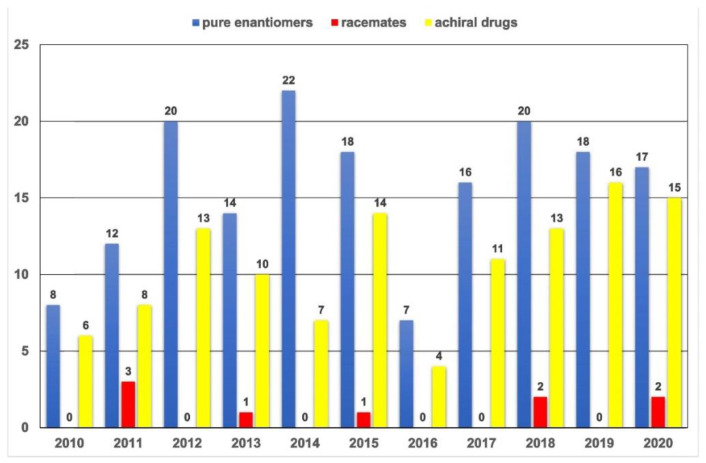
Comparison of the annual number of medications authorized by the FDA between 2010 and 2020 (pure enantiomers vs. racemates vs. achiral drugs).

The “chiral switch” concept appeared in the early 1990s and refers to the development of a pure enantiomer (the eutomer) from a previously approved and marketed racemate and is related to a change in its chirality status [8]. The chiral switch practice is sometimes considered to be controversial, especially in cases where the launch of pure enantiomer drugs derived from popular racemates had minimal therapeutic benefit over the racemate and it was used by pharmaceutical companies mainly as a patent protection tool against generic competition [9]. This approach was a significant component of drug development portfolios up to 2010; however, in the last ten years, new chiral chemical entities approved by the FDA have almost all been developed as de novo pure enantiomers [7,8]. The main routes for the de novo development of an enantiomerically pure drug are using a stereoselective synthesis (including enzymatic and biological procedures), chiral building blocks originating from natural products for the construction of the final molecule (“chiral pool”) and separating a racemate obtained by a non-stereoselective synthetic protocol (“chiral resolution”) [3]. Figure 1 displays the total number of pharmaceuticals the FDA authorized each year between 2010 and 2020 in accordance with three categories (single enantiomers, racemates and achiral drugs). It is noteworthy that throughout this time, the FDA authorized only nine racemates, and most approved drugs were single isomers [5].

Taking into consideration all the data mentioned above, the development of new analytical methods for the chiral separation of analytes is a challenge for the scientists. Among these enantioselective methods, liquid chromatography (LC), gas chromatography (GC), supercritical fluid chromatography (SFC) and capillary electrophoresis (CE) have been the most widely employed [10]. Even though LC has historically been by far the most popular enantioseparation method, CE has demonstrated some extremely intriguing properties for performing enantiomeric separations and has gained great importance and influence in the last decade in the chiral separation of pharmaceuticals [11,12].

**Figure 2:**
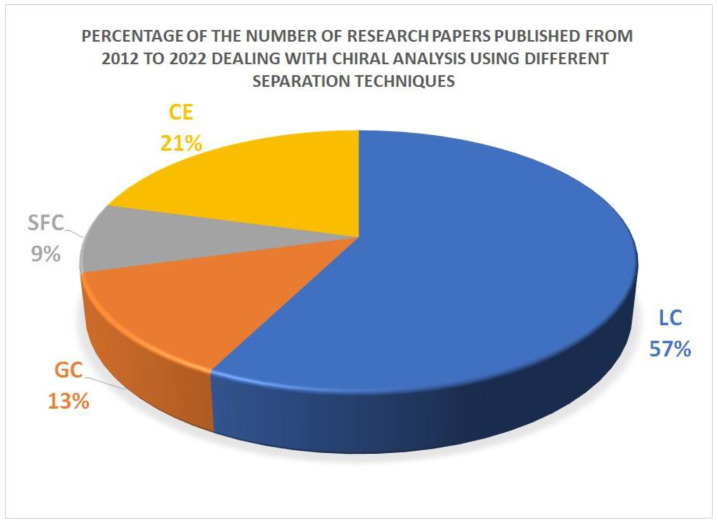
Percentage of research articles published from 2012 to 2022 (total number of articles 3230) dealing with chiral analysis utilizing various separation techniques, according to the Web of Science Reuters Clarivate Analytics database (Accessed 20 July 2022) (LC; liquid chromatography, CE; capillary electrophoresis, GC; gas chromatography, SFC; supercritical fluid chromatography).

To evaluate the use of CE in chiral analysis, we made a survey for the last 10 years on the Web of Science Clarivate Analytics database regarding the use of certain analytical techniques in the enantioseparation of chiral substances. The keywords employed were “chiral analysis” and “liquid chromatography”, “capillary electrophoresis”, “gas chromatography”, “supercritical fluid chromatography”. The results presented in Figure 2 show that CE is the second most frequently used chiral separation technique after LC.

Chiral analysis benefits from several of CE’s intrinsic qualities, including its high separation efficiency, quick analysis times and especially low reagent and sample consumption. In CE, usually a direct chiral separation method is used by adding the chiral selector (CS) directly to the separation medium, which confers chiral CE a high degree of flexibility because the type of CS (or combination of CSs) and its concentration can be changed with ease, facilitating the separation of enantiomers, and lowering the financial costs associated with the use of chiral chromatographic columns in LC [11,12]. The wide range of CSs (cyclodextrin derivatives, macrocyclic antibiotics, proteins, crown ethers, polysaccharides, ligand exchangers) available for use in CE further increases the popularity of this technique in achieving enantiomeric separation [13,14]. Additionally, the low quantities of chemicals, solvents and samples required in CE are well suited with “green chemistry” concepts.

The determination of the chiral purity of single enantiomeric drug substances is a big challenge for the analysts because the developed methods must enable the detection of a very low amount of the enantiomeric impurity (distomer) in the presence of the majority enantiomer (eutomer) [11]. Since there are no specific limit requirements for enantiomeric impurities, the general rules regarding drug impurities are applied. Therefore, according to ICH stipulations, the presence of the enantiomeric impurity cannot exceed 0.15% of the majority enantiomer in the case when the maximum daily dose is ≤2 g and 0.05% when the daily dose is >2 g [15]. This necessitates a separation with high chiral resolution to avoid overlapping of the majority peak and the enantiomeric impurity peak, as well as a high sensitivity to detect the enantiomeric impurity at such low concentrations. The enantiomer migration order (EMO) is another noteworthy element influencing the identification and quantification of the enantiomeric impurity; the distomer-first EMO is preferred, preventing the major peak from overlapping with the impurity peak [16,17,18].

Given the time-consuming nature of the “One Factor at a Time” (OFAT) strategies (where one analytical parameter is varied and the rest are maintained constant) for chiral separation method optimization, the use of Design of Experiments (DoE) strategies is highly recommended. The results of development studies, using DoE, can give insight into the relationship between analytical procedure (AP) variables (input factors) and AP replies (output responses). The implementation of Quality by Design (QbD) approaches is an important point for the development of chiral CE procedures suited for evaluating the enantiomeric purity of medications marketed as pure enantiomers. QbD involves defining the Analytical Target Profile (ATP) (for example, determining the main compound and its chiral impurity at the 0.15% level with high resolution in a short analysis time), identifying the Critical Method Parameters (CMP) (background electrolyte (BGE) concentration, BGE pH, CS concentration, temperature, voltage) influencing the Critical Method Attributes (CMAs) (chiral resolution, analysis time) and establishing Design Space (DS) (that reflects the experimental conditions under which the analytical target is reached) [19,20].

Soon after ICH published the ICH Q8 guideline [21], highlighting the importance of the QbD strategy for pharmaceutical development, analysts found that QbD principles could be applied to the development of analytical methods since it is also a “process” with quality standards that must be met; this resulted in what is now known as Analytical Quality by Design (AQbD). The applications of multivariate approaches (DoE) to method development are critical elements in the context of the AQbD initiative; these will probably be mandatory for analytical development in the future, especially after being adopted by regulatory bodies [22].

ICH guideline Q14 on AP development was released in March 2022. ICH Q14 [23] and ICH Q2(R2) [24] specify the development and validation activities that should be carried out during the lifespan of an analytical technique used to assess the quality of drug substances and medicinal products. ICH Q14 outlines the scientific principles for developing, managing, and submitting APs for the minimal and enhanced approaches, while ICH Q2(R2) specifies how to produce, submit, and preserve proof that an analytical technique is appropriate for the purpose (drug quality assurance). New ICH guidelines point out the importance of a comprehensive strategy in method development involving a multivariate approach and risk assessment.

The aim of the current review is to present an overview of the new perspectives, applying ICH Q14 and ICH Q2(R2) principles for CE method development for the control of the enantiomeric purity of drugs.

## 2. Recent Advances in the Development of CE Analytical Procedures for Enantiomeric Purity Control of Pharmaceuticals

When developing a CE AP suitable for the quality control (QC) of a drug product (DP) containing a single enantiomer Active Pharmaceutical Ingredient (API), two aspects are of outmost importance. First, the separation patterns of the analytes in the electropherogram can be easily modulated because the separation system can be finely and effectively tuned by changing BGE composition, CS type and operating mode. On the other hand, the optimized separation system should enable a robust separation of the enantiomers and the fulfilment of performance requirements of validation, so to assure that the AP performs adequately for the intended use. These two aspects merge when applying the innovative approaches for AP development recently outlined, leading to the integration between optimization and validation phases and ensuring a risk-based set-up of the AP [21,23,25]. These comprehensive approaches are founded on the pharmaceutical regulatory documents recently issued by the ICH, which propose a new quality paradigm for the development of highly reliable APs [21,23]. In this section, first a general description of QbD principles and their application to the development of an AP is described as outlined in regulatory documents. Secondly, some noteworthy aspects of recent applications in CE chiral separation of drugs are highlighted. Finally, more specific, and practical indications on the way of planning the development of a QbD-compliant CE AP for chiral analysis are presented.

### 2.1. Regulatory Requirements for Quality Purposes

Quality assurance (QA) is a major concern in the pharmaceutical industry. An in-depth knowledge of the quality of the DP released on the market is required for assuring its safety and efficacy to consumers. In recent years, the concept that quality should be designed and built in during the manufacturing process has been spreading all throughout the pharmaceutical scientific community. This represents a considerable shift from the traditional Quality by Testing (QbT) concept, where quality is simply checked during and at the end of the manufacturing process. This concept has been applied through the introduction of the QbD approach, involving a systematic methodology to pharmaceutical development. QbD was firstly adopted by the U.S. FDA [26,27], and shortly after ICH regulatory documents recommended the use of this innovative quality paradigm. Requirements for product quality are described in guidelines ICH Q8(R2) Pharmaceutical Development [21], ICH Q9 Quality Risk Assessment [28] and ICH Q10 Pharmaceutical Quality System [29]. In ICH Q8(R2), QbD is defined as “a systematic approach to development that begins with predefined objectives and emphasize product and process understanding and process control, based on sound science and quality risk management” [21]. Fundamental pillars of QbD are represented by quality risk management (QRM) and DoE. Guideline ICH Q11 Development and Manufacture of Drug Substances affords further clarification on the principles described by previous guidelines [15].

Concomitantly to the release of ICH Q8(R2) [21], the attention of the researchers in drug analysis was immediately directed to the application of QbD to AP development, creating the AQbD concept [30,31,32]. As a matter of fact, the APs are an integral part of pharmaceutical development and can be considered analogous to a manufacturing process. In this case the product is the AP itself, and the output is the Reportable Result (RR), defined by ICH as “the result as generated by the AP after calculation or processing and applying the described sample replication” [24]. The RRs support decisions on how development should be pursued and/or provide information on whether a DP should be released, thus their reliability is of fundamental importance [33]. AQbD concepts have been mainly addressed to separation techniques such as chromatography (LC, GC) and CE [34,35,36,37].

Several papers have been published in recent years focusing on redirecting and adapting the QbD paradigm to APs [25,33,38,39,40] and to show the benefits of AQbD in the QA of DPs [36,41,42,43,44,45,46]. AQbD is nowadays established as a systematic approach to AP development, following a well-defined framework, so to ensure that an AP is fit for its intended procedure over its entire lifecycle [33,47]. The contributions of both the academic and industrial research have paved the way toward the release of ICH guidelines Q14 Analytical Procedure Development [23] and Q2(R2) Validation of Analytical Procedures [24], with the purpose of adopting an analytical viewpoint in QbD.

### 2.2. ICH Q14 and ICH Q2(R2) Guidelines

Although it is not yet mandatory to adopt AQbD when developing a new AP, it is likely that soon, regulatory agencies will consider AQbD principles for new submissions [48]. Moreover, for a quality scientific paper in drug analysis, these concepts should be adopted in AP development [49]. The issue of ICH guidelines Q14 and Q2(R2), endorsed in March 2022 and currently under public consultation, is expected to address both the pharmaceutical industries and in general the scientific community to shift from the traditional OFAT AP development to this quality paradigm. The combined topic ICHQ2(R2)/ICHQ14 furnishes guidance on how to apply enhanced development approaches to APs and to define requirements for analytical validation [50].

#### 2.2.1. ICH Q14 Enhanced Approach

The objective of ICH Q14 [23] is to cover science and risk-based approaches for developing and maintaining APs suitable for the assessment of the quality of drug substance and DP. The guideline is strictly connected to ICH Q2(R2) [24] since not only validation data but also information on the development of an AP may demonstrate that the latter is suitable for the intended purpose. Two approaches to AP development are described: one “minimal” and one “enhanced”, which includes other elements in addition to the minimal. The second one includes more robust procedures, better understanding of the impact of AP parameters and more flexibility for lifecycle management [23].

**Figure 3:**
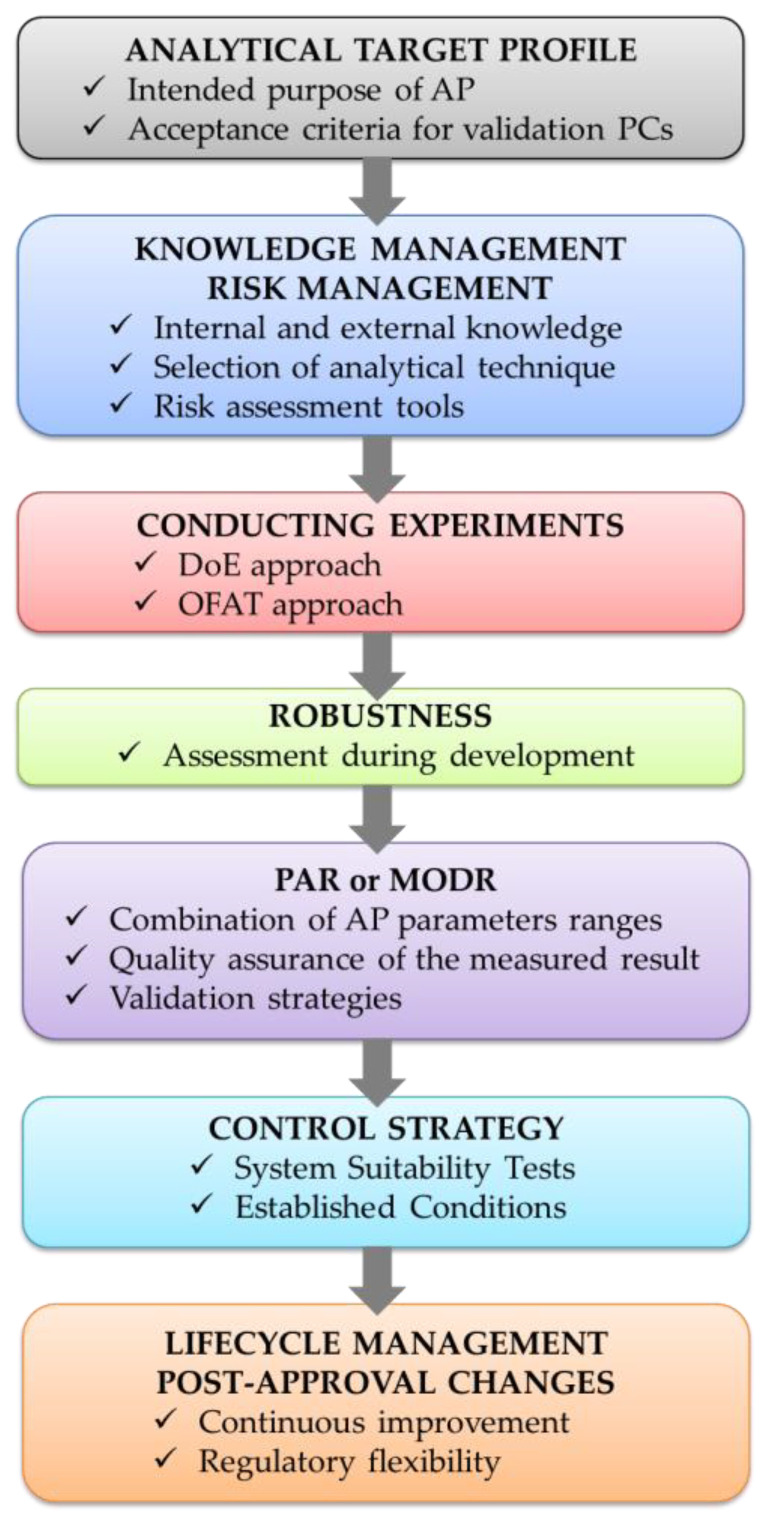
The key steps of ICH Q14 enhanced approach.

In Annex A, one example concerns the measurement of stereoisomers as process-related impurities in a small molecule drug substance. Hence, its consultation may be very useful for approaching the determination of the enantiomeric purity of a drug substance/DP. In this section we focus only on the most general aspects of the enhanced approach, including: (i) Analytical Target Profile (ATP); (ii) Knowledge management and risk management; (iii) Conducting experiments; (iv) Robustness; (v) Proven Acceptable Ranges (PAR) or Method Operable Design Region (MODR); (vi) Control strategy; (vii) Lifecycle management and post-approval changes. In Figure 3 the key points of the enhanced approach are outlined.

(i) *Analytical Target Profile*: The ATP consists of a description of the AP’s intended purpose and includes the performance requirements of the measurements, as well as acceptance criteria for performance characteristics (PCs) of the AP’s validation. The ATP is not related to a particular analytical technology [45].

(ii) *Knowledge Management and Risk Management*: Prior knowledge can be internal, namely from experience in the laboratory, or external, such as reference to scientific publications, and is also important for selecting a suitable analytical technique. QRM uses tools shown in ICH Q9 for defining the AP parameters with potential impact on the performances, for prioritizing them and for establishing the possible entity of the impact [28].

(iii) *Conducting experiments*: The enhanced approach consists of carrying out OFAT or multivariate experiments to investigate the effects of the AP’s parameters on the performances. The univariate approach is still included in the enhanced approach; nevertheless, the use of DoE leads to several advantages: an in-depth knowledge through a limited number of experiments, the detection of interactions between factors, the calculation of mathematical models linking responses to factors and the simultaneous optimization of more responses [51,52,53,54].

(iv) *Robustness*: Robustness should be evaluated during development and is defined as “a measure of the capacity of the AP to meet the expected performance requirements during normal use” [23].

(v) *Proven Acceptable Range or Method Operable Design Region*: Depending on whether the experiments to investigate AP parameters are carried out in a univariate or multivariate way, PAR or MODR are established, respectively. In the enhanced approach, DoE makes it possible to obtain wide knowledge on the effects of the AP’s parameters on performances by Response Surface Methodology (RSM) [51,52,53,54] and then to identify an MODR. The latter is defined as “a combination of analytical procedure parameter ranges within which the analytical procedure performance criteria are fulfilled and the quality of the measured result is assured” [23]. Moving within the PAR or the MODR does not require regulatory notification. Moreover, only the part of a PAR or an MODR that is intended for routine use must be covered by validation data. In Annex B two strategies are mentioned for validating the MODR, with the possibility of in-between options: validation can be performed at a single set of operating parameters or at the working point and at the edges of the MODR.

(vi) *Control Strategy*: An analytical control strategy should ensure that the AP performs as expected during routine use and it consists of a series of controls derived from development data, risk assessment and a robustness test. It should be defined before validation, including AP parameters needing control and system suitability tests (SSTs). The SSTs are employed to verify that the AP measurement system and the analytical operations are adequate and increase the detectability of potential failures. Established Conditions (ECs) consist of legally binding information considered necessary to assure product quality; they may be performance criteria, but also sets of points or ranges for the AP parameters.

(vii) *Lifecycle Management and Post-Approval Changes*: Post-approval changes to the AP can occur, often driven by continual improvement [43,47,48]. In the enhanced approach, a lifecycle management plan is defined, reporting categories as the ECs, the PAR or the MODR. If an EC is changed, a submission to the regulatory authority is needed. On the other hand, only changes outside the approved range of PAR and MODR require regulatory reporting, with higher regulatory flexibility [55,56].

#### 2.2.2. ICH Q2(R2) Outlook

The ICH Q2(R2) guideline consists of a complete revision of the ICH Q2(R1) guideline [24], to include the more recent application of APs and to align content with ICH Q14 [23]. Only the main points that have been modified and that can impact on the development of CE APs will be presented in this context. What is new is that suitable data from development studies can be used in the place of validation data with appropriate justification [24]. Moreover, SSTs are a part of the AP description and are generally established during development.

According to the intended use of the AP, the product attributes are described as (i) Identity; (ii) Impurity (Purity), Quantitative or Limit; (iii) Assay content/Potency. The objectives of the AP are described by PCs (previously called Validation Characteristics), listed as Specificity, Working Range, Accuracy and Precision, with the related Performance Criteria. Hence, the number of primary PCs has been reduced with respect to ICH Q2(R1), and the former “Linearity” and “Range” have been replaced by “Working Range” for giving the possibility of using any type of calibration model [57].

Another novelty regards the validation during the AP lifecycle. If changes in the AP are needed, a full or partial revalidation could be required. Anyway, science and risk-based principles can be used to justify whether a PC needs revalidation, thus further encouraging the enhanced approach to be embraced.

Some changes in the validation tests deserve attention:

- The term “Selectivity” is placed beside “Specificity”, covering the common possibility of uncomplete discrimination of the analytes, but at the same time giving value to the minimization of interference, even if the method cannot be proved as “specific”;
- The Lower Range Limits, Detection Limit (DL) and Quantitation Limit (QL), are now included into the PC “Working Range”. In the case of impurity testing, when building the curve for assessing the relationship between analyte concentration and response, the lower concentration value of the Working Range should correspond to the QL;
- As concerns Accuracy and Precision, there is the possibility of considering their total impact instead of evaluating them separately. The concept of Total Analytical Error has been mentioned, together with the AQbD approach, as a factor necessary for a quality scientific paper in drug analysis [46,49]; as a matter of fact, the uncertainty of the RR is related to Total Error;
- For assessing Intermediate Precision, the variations tested (days, environmental conditions, analysts, equipment) should be based on AP understanding from development and risk assessment.

### 2.3. Capillary Electrophoresis for Enantiomeric Purity of Drugs

Electromigration techniques, performed in capillary or microchannel format, are inherently miniaturized with the advantage of requiring small amounts of sample, thus are suitable when separations at an analytical scale must be performed. In the electrokinetic mode (electrokinetic chromatography, EKC), the analytes are separated under the application of an electric field, through a capillary filled with BGE solution. Lacking the stationary phase, the EKC separations are driven by the differences in electrophoretic mobility of the analytes.

Chiral separations can be accomplished either by the direct or indirect approach [6,58,59]. The latter consists of the derivatization of the analytes’ enantiomers with a pure chiral reagent able to yield diastereomers, which, having different physicochemical properties, are endowed with different electrophoretic mobility, and can be separated without the use of a CS [60,61,62,63]. Chiral derivatization can be advantageous especially when the reaction yielding the diastereomers is fast and occurs under mild conditions, avoiding analyte degradation. The compatibility of the chiral reagent with aqueous media is a further important point since most of the target chiral analytes are found in aqueous matrices, e.g., biological and environmental samples, and CE separations are mainly carried out in aqueous BGEs [20,64,65,66]. The compound 1-(9-fluorenyl)ethyl chloroformate (FLEC) as a pure enantiomer i.e., (+) or (−)-FLEC, is an example of a useful chiral derivatizing reagent for the indirect enantioresolution of primary and secondary amines through the formation of stable and non-racemizing derivatives. Once the diastereomers have been obtained, the optimization of their CE separation can span a wide range of conditions including some constrained ones such as those necessary for MS hyphenation [61]. In the case of a very fast reaction between analytes and the derivatizing reagent, as achieved using *ortho*-phthalaldehyde and *N*-acetyl-*L*-cysteine, the in-capillary chiral derivatization can be performed, limiting the sample handling, and making the process automatable [67].

#### 2.3.1. Chiral Selectors in EKC and Separation Mechanisms

The most frequently used approach in chiral electromigration techniques is the direct method, where a CS is supplemented to the BGE enabling enantiodiscrimination through the ability of the in situ formation of transient diastereomer complexes during electromigration. In this regard, the miniaturized format of EKC presents an additional advantage because reagent consumption, including the CS, is limited [11,13,58,59]. Direct enantioseparation in EKC can occur by two possible mechanisms: differences in the binding constants of the two enantiomers with the CS and differences in the mobilities of the transient diastereomeric complexes, although CE enantioseparations are in most cases dominated by the former [58]. Several studies have been addressed toward the comprehension of the analytical enantioseparation processes; the mechanisms involved in enantiodifferentiation depend on the nature and the characteristics of the CS; however, in many cases they are not fully understood because of the complexity of the selectors [68]. The various CSs used in EKC can be classified as macromolecules (e.g., cyclodextrins (CDs), linear polysaccharides including glycosaminoglycans and maltodextrins, proteins), supramolecules (e.g., chiral surfactants able to form chiral micellar aggregates) and ligand exchangers as well as other small molecules (e.g., chiral ionic liquids (ILs)) [13].

CDs are the most successfully used CSs in CE due to their wide variety (native and derivatized, neutral and charged), UV transparency, good solubility in most BGE used in CE, limited toxicity and generally high enantioselectivity due to complexation by inclusion [69,70,71]. Since enantioseparation using CDs is not based on the phase distribution equilibria, affinity capillary electrophoresis (ACE) may be a preferable term for this kind of method [72,73]. ACE is also involved in chiral separation using proteins as CSs; the enantiorecognition by proteins is a complex phenomenon involving hydrophobic as well as coulombic and hydrogen bond interactions with the chiral molecule. The inherently chiral nature of proteins is conveniently preserved when they are supplemented as CS into the BGE instead of immobilized on HPLC or CEC supports. The drawbacks of this approach, as the adsorption of the protein to the capillary wall, and their UV absorbance at the detection wavelength, have been tackled using coated capillaries and by partial filling with the protein BGE up to the optical window of the capillary, respectively [74].

Chiral surfactants have been effectively applied in chiral separations by CE in MEKC mode either alone or as additives to improve enantioseparations by other CSs. As a representative example, bile salts are able to form chiral micelles whose aggregation architecture, which depends on the surfactant concentration, influences the chiral discrimination ability [13]. The enantiorecognition by chiral micelles is not related to complexation phenomena as in ACE since the distribution of the analytes into the micelle is driven by the retention factor (the ratio between the amount of the analyte in micelle vs. bulk phase) [75].

In the chiral ligand exchange mechanism, the enantioselectivity is achieved via the difference between the thermodynamic stability of the ternary complexes formed between analytes, a central metal ion (e.g., Cu(II)) and chiral ligand, often represented by an enantiomerically pure amino acid. Only limited applications of the method are given in the field of pharma analysis [76,77].

#### 2.3.2. Selected Examples of Chiral EKC of Drugs

In this section we mention some examples of EKC chiral separation of drugs, which deserve particular interest regarding the recent trends in the use of CSs and/or in the studies of separation mechanisms, referring to recent years.

A model application on how the enantioresolution can be achieved based on differences in the mobility of the transient diastereomeric complexes is given by the chiral purity determination of the immunomodulatory agent S-lenalidomide (at 0.1% level of the R-distomer) using sulfobutylether-β-cyclodextrin (SBE-β-CD) as the CS [78].

A further feature of the chiral separation in CE with respect to chromatography is the wide opportunity to control the EMO by the proper selection of the CS (nature and concentration) and operative parameters (e.g., BGE pH) [71]. Tuning the EMO is of utmost importance in pharmaceutical analysis since the main component (the eutomer) can overlap the minor component due to peak tailing. Thus, generally, a distomer (enantiomeric impurity) first EMO is preferred in chiral analysis. In this way, also in the case of the overloading necessary for the analysis of the distomer at impurity level, the detectability of the latter will not be compromised by the eutomer peak tailing. In a recent application, the chiral purity of *R*-solriamfetol, a novel norepinephrine–dopamine reuptake inhibitor, was established both in API and related pharmaceutical preparation, at the level of 0.1–1.2% of the distomer *S*-solriamfetol, by using sulfated-γ-cyclodextrin (S-γ-CD) as the CS [79]. Interestingly, EMO reversal was obtained moving from the highly sulfated randomly substituted CDs to the single isomers, representing an effective example of how subtle differences in CD structure and composition can alter the recognition pattern [70,79]. By properly adjusting the pH, EMO reversal can be achieved either by using neutral [80] or negatively charged CDs [81]. In the quantitation of *S*-rasagiline as the distomer of *R*-rasagiline antiparkinsonian agent, the combined selection of the BGE pH value with the application of an external pressure during the electrophoretic run, has been shown as a strategy for manipulating EMO and reducing analysis time. By using SBE-β-CD as CS at pH 2.0 to strongly suppress the electroosmotic flow (EOF), the desired EMO (the distomer migrating faster) was obtained and the application of a weak external pressure (8 mbar) allowed the analysis time to be shortened with a concomitant improvement in peak shape, obtaining a QL of the distomer of 0.15% [82]. In a study for the enantiomeric quality control of *R*-praziquantel, EMO was reverted by a simple polarity switch (detection end at anode), and suppression of EOF, resulting in increased resolution and migration times (reduced by employing short-end injection) [83].

As well as the development of strategies and technical approaches to improve chiral separations, a considerable number of investigations have recently been carried out to better understand the intriguing mechanisms of enantiorecognition by CDs. The combination of electrophoretic data (enantioselectivity, resolution, mobility) with nuclear magnetic resonance (NMR) spectroscopy and molecular modeling have disclosed the role of the CD cavity size and substitution pattern on EMO [71,84,85,86,87,88,89,90,91]; interestingly, CE was shown to be a very sensitive technique to study the affinity patterns in CD complexes with chiral guests [71,84], whereas NMR and molecular modeling approaches allowed hypotheses to be advanced regarding the spatial structure of selector–analyte complexes. Recently, it was also shown by the group of Chankvetadze that inclusion complexation is not a prerequisite for CD-based enantioseparations, and that enantiodiscrimination can also be achieved through the formation of shallow, external complexes [92,93]. To understand the effects of substituents and their position on enantiodiscrimination, and to gain information on the molecular recognition processes, the investigations cited above have involved single isomer CDs instead of the more easily available randomly substituted ones. The use of single isomer CDs can also be justified from a practical point of view as they are less prone to the batch-to-batch variability than the widely used mixtures of isomers [69,81].

One of the distinguishing features of chiral separations in CE is the possibility of extending enantioselectivity through the synergistic effect of multiple chiral selectors supplemented into the same BGE; in this regard, the use of dual CDs systems has become quite common [70,94,95]. Together with the well-known approach where mixtures of different CDs are simultaneously dissolved into the BGE for their synergistic effect (usually one is an anionic CD and the other is a neutral one), two CDs showing different enantioselectivity toward the analytes can be introduced in the capillary as two distinct sample plugs in a dual selectors partial filling mode [96].

The use of additives to enhance/modulate CD-driven chiral separations has been known for a long time, and CD-modified micellar electrokinetic chromatography (CD-MEKC) [97,98] as well as CD-modified microemulsion electrokinetic chromatography (CD-MEEKC) [99] can be included in this strategy. Recently chiral ILs have been the focus of an intense interest as additives for their synergistic effect on CD-driven enantioseparations [100,101,102,103,104,105,106,107,108,109,110,111,112], and in some instances, for functionalization of the CDs [113,114]. The synergistic effect on the chiral separation by CDs has recently been reported by the utilization of eutectic solvents [115,116,117].

Chiral CE method development includes several steps; the main factors to be optimized are the type and concentration of the CS. In this regard, in the case of CDs, the mobility difference model establishes that separation selectivity could be expressed as a function of the selector concentration by assuming that the analyte is completely ionized under separation conditions [118,119]. Because of the importance of the pH of the BGE, the charged resolving agent migration (CHARM) model was introduced [120]. From the practical point of view, in most of the applications for chiral purity determination, the selection of the optimum conditions for each of the relevant parameters is driven by the analyst’s experience and by the relevant physico-chemical information of the analytes, e.g., pKa, logP, UV absorption spectra, etc. Then, the magnitude of each of the factors is changed, while the others are kept constant to establish their influence on the separation to find the optimum conditions. This conventional strategy, defined as a univariate approach (OFAT), is, however, increasingly being replaced with DoE, allowing for a better evaluation of the multidimensional effects and interactions of the input factors on the output responses of the CE method, which result in a fewer number of experiments required for optimization and deeper method understanding [19,121].

Table 1 and Table 2 present a selection of CE chiral purity studies of several pharmaceuticals published in the last 10 years employing DoE and QbD, respectively, for method development. The literature survey for the tables below was made on Google Scholar between 2012 and 2022 on CE methods, which employed multivariate approaches to develop and consolidate chiral purity analysis methods. The articles are listed in the tables chronologically.

### 2.4. AQbD Framework in the Development of CE Analytical Procedures

**Figure 4:**
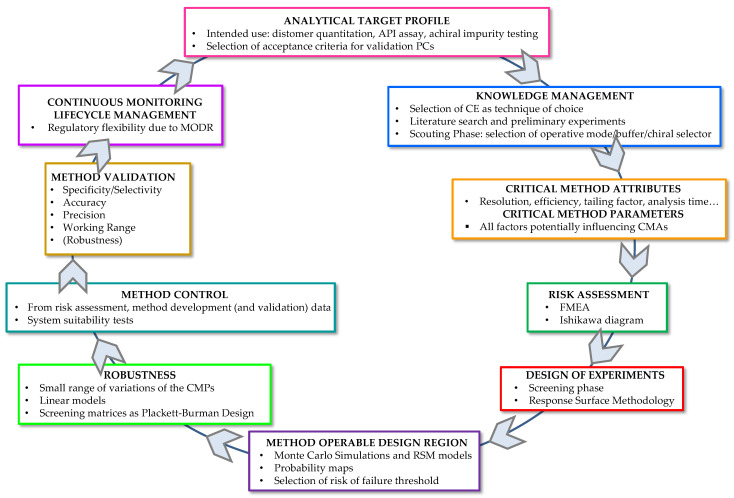
Analytical Quality by Design in CE.

The application of the AQbD approach in the development of CE APs for the evaluation of the enantiomeric purity of drugs marketed as single enantiomers should be recommended, as it represents a clear trend of drug analysis. The AQbD strategy in this field has been applied in a few cases (Table 2) [129,130,131,132,133,134,135,136,137,138,139,140] but deserves a wider attention. In this section a brief description of the AQbD compliant development of a CE AP is proposed, highlighting only the relevant information. The AQbD framework is schematized in Figure 4, highlighting the fact that AQbD is a circular concept.

#### 2.4.1. Analytical Target Profile

The intended purpose of the AP can be defined as the quantitation of the distomer in the eutomer API for release testing. The AP should allow the quantitation of the distomer to verify the enantiomeric purity ≥ 99%, with a baseline resolution of the enantiomers and a reasonable analysis time, depending on the analytical task. If the method is intended to be used for assay and quantitation of the API and other achiral impurities also, this should be specified in the ATP too. Validation PCs should include Specificity/Selectivity, Accuracy, Precision and Working Range, all of them with their acceptance criteria. Some of the acceptance criteria could be listed as: QL for the enantiomeric impurity ≤ 0.15% (*w*/*w*) with respect to the main compound, with average recovery included in the range 90–110% and intermediate precision RSD ≤ 10%. As for Specificity, the lack of potential interferences with quantitation of the impurity by other components in the sample is required, and a Working Range, from the reporting threshold to 120% of the specification limit, should be verified [23,141]. This is only a mock example of how an ATP could be structured: more details can be found in ICH Q14 Annex A and could be adapted to the intended use of the AP [23].

#### 2.4.2. Knowledge Management

Once the ATP has been defined, an analytical technique able to fulfil the ATP requirements should be selected. CE is clearly among the techniques of choice for determining enantiomeric purity due to its great advantages already described in Section 2.3. The selection of CE can be widely justified based on the ATP, considering the desired performances and practical criteria such as time and costs of analysis. Knowledge management is then performed, and, in this context, an experimental scouting phase is carried out to find the suitable CE operative mode and to identify the separation system, including the BGE components, the type of pseudostationary phase if present, the type of CS, or at least a selection between a limited number of CSs to be further investigated by the subsequent multivariate approach. The scouting phase deserves much attention because it will address the general conditions of analysis, which will be extensively tuned and investigated by DoE [35]. It may be advisable to perform the scouting experiments with a test mixture containing the single enantiomer API at a low concentration value, such as the distomer, to better understand how the separation pattern changes when varying experimental conditions and to identify which analytical conditions can possibly lead to a robust separation [136].

#### 2.4.3. Critical Method Attributes and Critical Method Parameters

The next step is the choice of the measured responses for controlling the performances of the AP and for reaching the ATP, namely the CMAs. A robust separation between the eutomer and the distomer is required; hence, enantioresolution (R_s_) or separation criterion (S) will be, in most cases, included among the selected CMAs for obtaining a satisfactory selectivity [35]. Nevertheless, many other CMAs can be defined depending on the analytical task, for instance, the efficiency or the tailing factor of the peaks, as well as the analysis time or the generated current. In addition, other types of CMAs could be linked to validation PCs: the peak area can be used to decrease DL and QL values, and the standard deviation of the peak area can be chosen to improve repeatability.

All the AP parameters that could have a potential influence on the CMAs should be identified and investigated. Thus, it follows that the number of CE parameters that are involved in AP development can be very high [19,142]. These are called CMPs and the subsequent risk assessment has the purpose of helping the researcher to define which of them could be fixed directly and which of them should be in-depth studied by DoE methodologies.

#### 2.4.4. Risk Assessment

According to ICH Q9 guideline, risk assessment consists of the identification of hazards and of the analysis and identification of risks associated with exposure to those hazards [28]. Several risk assessment tools are mentioned, among which there are Failure Mode and Effect Analysis (FMEA) and Ishikawa (fishbone) diagram [28]. In the FMEA method, a quantitative risk assessment is performed, furnishing a Risk Priority Number (RPN) estimated by the product RPN = PxSxD, where P is the occurrence probability, S is severity and D is the likelihood of detection. CMPs with an RPN above a threshold level should be further studied, while those with a lower RPN can be fixed [22,31]. Instead, in Ishikawa diagrams, a qualitative risk assessment is performed, where the CMPs are classified in categories.

**Figure 5:**
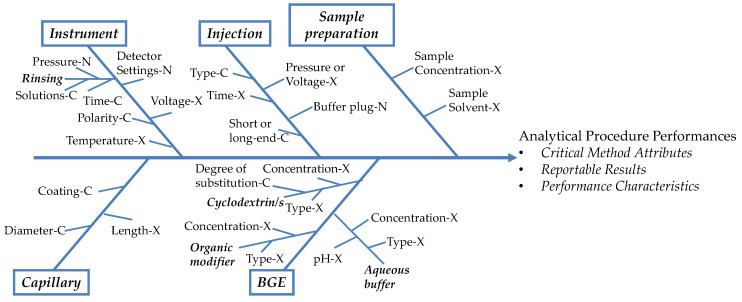
Ishikawa diagram for a generic CZE method. Analytical procedure parameters: C—controlled factor, N—noise facto, and X—experimented factor.

Figure 5 shows an example of an Ishikawa diagram for a generic CZE method for the determination of enantiomeric purity, using CDs as CSs.

The AP parameters can be classified into main categories: instrument, injection, sample preparation, capillary and BGE. Among the AP parameters, the researcher defines which should be controlled (C), which are noise factors (N) and which should be experimented (X), with the CMPs represented by the C- and the X-factors [30,136]. Among the CMPs that should be investigated, some can be fixed by performing preliminary/scouting experiments, often including capillary type and length, injection time and pressure, sample concentration, rinsing time and pressure, CS type and BGE type. Other CMPs are mostly fixed after performing a DoE study, including CS concentration, BGE concentration and pH, organic modifier concentration and voltage, depending on the results of screening experiments and of QRM. In principle, capillary length, CS type and buffer type could also be investigated in a screening step as qualitative factors. The AP performances include the CMAs and can also be related to the validation PCs, for instance, the peak area at concentration values near the QL for evaluating sensitivity, the repeatability of migration times and corrected peak areas and so on.

#### 2.4.5. Design of Experiments

**Figure 6:**
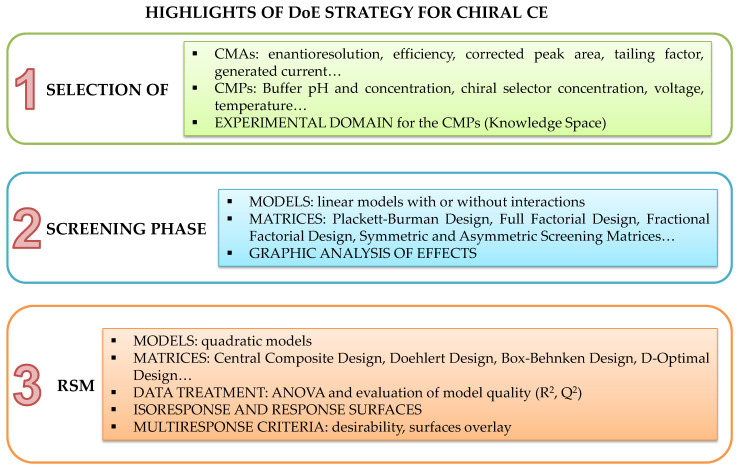
DoE strategy for chiral CE.

Even if the OFAT approach for investigating AP parameters is accepted by ICH Q14, the use of DoE should be widely encouraged [23,52,54,143]. Several reviews have been published on the use of DoE methodologies in the development of CE methods [19,121,142,144] and a full description of DoE strategy falls beyond the topic of this paper. More information can be found in the above-mentioned dedicated reviews, and here only some basic indications are furnished, schematized in Figure 6.

DoE is a systematic approach for the investigation of phenomena depending on more factors, which are simultaneously varied according to matrices enabling the study of the effect of the factors on one or more responses. Polynomial models’ (linear, linear with interactions and quadratic) linking factors to responses are hypothesized. A suitable design for estimating the model coefficients is selected considering the peculiar features of each matrix in terms of the number of experiments required, type of model that can be calculated, and the efficiency and possibility of fractionating the experimental domain [51,52,53,54,143].

Usually, a first screening step is performed in an experimental domain, called knowledge space, which is the zone selected based on preliminary/scouting experiments leading to the fulfilment of the requirements for the AP performances. The aim of screening designs is to investigate the effects of a high number of factors with a low number of experiments. All the potential CMPs are considered, both qualitative and quantitative, and linear models with or without interactions are generally hypothesized. Typical analytical parameters that are usually studied include BGE concentration and pH, CS type, second CS type, ratio between a pair of CS, voltage, and temperature. Screening matrices such as Plackett–Burman design (PBD), full factorial design (FFD) or fractional factorial design (FrFD), as well as symmetric and asymmetric screening matrices, are able to supply adequate information with a limited number of experiments. All of these allow an informative graphical representation of the effects, making it possible to fix the values of the CMPs that exert no significant influence on the AP performances, or whose effect is well defined [51,52,53,54,143].

Then, a more in-depth investigation can be performed by subsequent RSM, in which the CMPs are studied at more than two levels and the relationship to the responses is described by a quadratic polynomial model; in this way it is possible to evaluate the model curvature. Usually, a central point is replicated to have an estimate of the experimental variance, thus allowing the validity of the model to be assessed. Some of the most used designs are the Box–Behnken design (BBD), central composite design (CCD), Doehlert design (DD) and D-optimal design. A mixture process variable approach can also be employed when both the mixture and process variables are involved in the optimization of the AP. By application of ANOVA (Analysis of Variance) the statistical significance and the lack of fit of the model are evaluated [52,53]. Other quality parameters of the models are also calculated, namely the coefficient of determination R^2^ and predicted variation Q^2^, and values of R^2^ > 0.8 and Q^2^ > 0.4 are needed for acceptable model fitting [54]. If the model is valid and the quality parameters are good, it is possible to draw isoresponse or response surfaces, achieving a detailed description of the predicted responses all throughout the investigated domain. This marks the difference from the traditional OFAT approach, as the latter limits the gained knowledge only to the tested points. Moreover, multiresponse criteria tools can be used simultaneously to optimize more responses, such as desirability and response overlay [51,52,53,54,143].

#### 2.4.6. Method Operable Design Region

To establish the MODR, it is necessary to consider not only the predicted average values of the CMAs from the models, but also the probability that the CMAs requirements are fulfilled. This can be performed by means of different methodologies, including Bayesian modelling, bootstrapping techniques, or Monte Carlo simulations [34,36]. Up to now Monte Carlo simulations [145] combined with the RSM models has been the common approach used in the case of CE methods for chiral separations [129,130,131,132,133,134,135,136,137,138,139,140]. By using this methodology, model error is included in the predictions of the response distributions and the risk of failure of exceeding the CMAs’ desired limits is calculated [54,145]. Probability maps can be drawn and the MODR can be defined by specifying a selected level of probability that the requirements for the CMAs are fulfilled.

#### 2.4.7. Robustness

It is worthwhile noting that the MODR can be assumed as a zone of robustness just for its ICH definition because “within its edges the AP performance criteria are fulfilled, and the quality of the measured result is assured” [23]. Nevertheless, some further testing on robustness may be advisable because some parameters may have been optimized and fixed early in AP development, in the screening phase or even in the scouting phase/preliminary experiments. Consequently, no information on the effect of their changes on the AP performances has been collected when performing RSM. When conducting a robustness test, due to the small interval of the CMPs studied, linear models can be hypothesized [19,35]. Typical examples of investigated CMPs may include selected X-factors and/or C-factors in the Ishikawa diagram, if they can be modified in a small range. One of the most used designs is PBD, where the CMPs are studied at two levels and the number of experiments is kept to a minimum [19].

#### 2.4.8. Method Control

The control strategy of the AP can be derived from the data collected during method development, including risk assessment, and method validation and consists of a planned set of controls that ensure that the AP performs as intended [35,56,146]. An example is the definition of SSTs from the results of robustness testing or instrumental repeatability studies [35]. Acceptance intervals can be indicated for analysis time, enantioresolution value, peak efficiency, peak tailing, generated current, or other AP performance parameters, that are monitored to assure that the RRs are reliable.

#### 2.4.9. Method Validation

For the determination of enantiomeric purity, a focus should be made on the product attributes’ impurity (quantitative) and assay content. In this context, in ICH Q2(R2) Annex 2 [24], a comprehensive explanation is provided on how to perform validation studies involving separation techniques, including CE, for quantitative determination of the impurities and assay of the API. A scheme of all the PCs and the related validation study methodology is reported. This, in detail, includes: (i) Specificity/Selectivity; (ii) Precision as Repeatability and Intermediate Precision; (iii) Accuracy; (iv) Working Range; (v) Robustness, performed as a part of AP development. The main difference with respect to the traditional AP development is that in AQbD the validation PCs and their acceptance criteria are described in the ATP [56].

#### 2.4.10. Continuous Monitoring—Lifecycle Management

AQbD involves a circle process and not a simple linear framework, as outlined in Figure 4 [36,56]. As a matter of fact, once validation is completed, AP performances should be continuously monitored to ensure that the AP remains in compliance with the ATP [55,146]. This can be performed for instance by using control charts or tracking system suitability data to quickly identify and address out of trend performances [55]. Moreover, the MODR allows flexibility for continuous improvement in AP [56]. This section is of paramount interest for the industry applicant, but usually deserves lower attention from the academic researchers, who are mainly involved in the set-up of APs more than their long-term routine use for assuring DP quality.

## 3. Conclusions

The development of effective and robust methods for the chiral separation of enantiomers is essential for studying the chiral purity of chiral substances used in therapy in the form of single enantiomers, to assure their quality.

Traditionally, optimization of analytical methods was carried out using an OFAT approach, and although this strategy may yield the most appropriate condition, it seldom yields an estimate of the “real” optimum condition.

CE is a powerful analysis method that can be applied for determining the enantiomeric purity of pharmaceuticals. It is noteworthy that there is an increasing interest in using it, resulting, statistically, in it being the second most frequently used chiral separation technique in the last 10 years after LC.

The application of DoE strategies is favorable for optimizing enantioresolution approaches using CE. The multivariate evaluation of the factors that impact enantioresolution reveals the method’s behavior under different analytical settings. Given the advantages of using DoE, it is surprising that this strategy has not been widely used in the development and optimization of CE enantioselective methods.

QbD aims to ensure that all sources of variability affecting a process are identified and managed using appropriate measures. The application of QbD to APs (AQbD) should lead to a well understood, robust and fit for purpose method throughout its lifecycle. AQbD is a structured method development approach that focuses on clarifying a method’s properties as its variables change so that it may be managed by scientific evidence and risk management. Although the AQbD approach at first glance seems to be more complicated than the classical QbT, it offers some indisputable advantages over the latter one, recognized by regulatory agencies as well. QRM and DoE can help to plan and execute AP development, obtaining MODR and providing quality assurance based on risk evaluation and additional flexibility for its application in the routine analysis.

Although the application of AQbD is beneficial for CE chiral methods’ development and optimization, it has received relatively little attention in the development of CE-based enantioselective techniques. The literature survey (2012–2022) we conducted on Google Scholar, Web of Science and Scopus indicated the publication of fewer than 15 articles that applied AQbD to develop and consolidate CE chiral separation methods. This may be due to the specific risk-based approaches and nomenclature of AQbD that are unfamiliar to many researchers, as well as the need for basic knowledge regarding DoE methodologies. However, as regulatory guidelines have recognized the importance of the new quality paradigm in AP development, it is expected the AQbD concept will gain more popularity in the scientific community.

The first stage of AQbD is the selection of an appropriate ATP, which should provide the AP’s performance characteristics and related criteria to guarantee that the resulting data are appropriate for the intended purpose. An ATP could indicate, for example, that a chiral impurity should be measured at a specified level in the presence of the API with good precision and accuracy in a short analysis time. The development of a conventional chiral CE method begins with a preliminary exploration phase in which an appropriate CS is identified and the experimental parameters to be evaluated, as well as their ranges, are chosen. Further development includes the identification of CMPs, which impact the CMAs of method performance. CMPs that are frequently considered include BGE concentration, BGE pH, CS concentration, temperature, and voltage, but also additional parameters such as the content of the organic modifier or injection conditions are sometimes used. Chiral resolution and migration time of the last migrating enantiomer are considered the main CMAs; however, sometimes separation factor is also used, or characteristics related to peak shape as well, such as efficiency. During the screening and optimization phases, CMAs are often the same. FrFD, FFD or screening matrices are usually employed for the identification of the critical parameters. Once the CMAs’ target values are specified, the significant parameters are subsequently optimized usually using FCCD designs and Monte Carlo simulations to define the MODR, which refers to the range of experimental parameter settings where the ATP is fulfilled with a selected probability. PBD is used to test robustness, and all established procedures can be validated in accordance with ICH recommendations.

ICH Q14 is intended to improve regulatory communications as well as facilitate sound scientific, risk-based approvals and post-approval change management. APs developed and validated by applying the new guidelines ICH Q14 and ICH Q2(R2) have much greater flexibility, as well as consistently deliver the intended performance throughout their lifecycle. The method can be validated in a single set of parameters from MODR, or a set point and the extrema parameters of MODR; thus, in the latter case it allows full operational flexibility within the entire range without further validation. Moreover, other in-between solutions may be chosen. ICH Q14 and Q2(R2) highlight AQbD as an enhanced approach, while the validation process is strongly connected to the AP development since both demonstrate the suitability of AP for its intended use; for example, data obtained during the development stage can be used in the validation as well.

## Figures and Tables

**Table 1:** CE methods for the chiral purity analysis of pharmaceuticals optimized by using DoE (2012–2022).

CE Technique	Pharmaceuticals	Matrix	DoE Methodology Factors/Responses	Optimized Analytical Conditions	Reference
CZE-LIF	Magnesium-l-aspartate (chiral impurity: d-aspartic acid)	bulk substance, tablets	Screening FFD BGE concentration, BGE pH, HP-β-CD concentration/chiral resolution *D*-aspartic acid impurity determination at 0.06%	50 mM phosphate BGE, pH 7.0, 18 mM HP-β-CD, 18 mM (*v/v*) DMSO, 20 kV, 25 °C	[122]
CZE-UV	Vildagliptin (chiral impurity: *R*-vildagliptin)	bulk substance, tablets	OD BGE concentration, BGE pH, SBE-α-CD concentration, temperature, voltage, injection parameters/chiral resolution Robustness testing: PBD *R*-vildagliptin impurity determination at 0.43% level	75 mM Tris-acetate BGE, pH 4.75, 20 mM SBE-α-CD, 25 kV, 15 °C, 200 nm	[123]
MEKC-UV	Montelukast (chiral impurities: *R*,*R*-*trans*-montelukast, *R,S-cis*-montelukast, *S,R*-cis-montelukast)	bulk substance, chewable tablets, oral granules	OFAT BGE concentration, TM-γ-CD concentration, SBE-β-CD concentration, temperature Screening FFD BGE pH, voltage/chiral resolution Robustness testing: PBD *R,R*-*trans*-montelukast impurity determination at 0.02% level	20 mM borate BGE, 10 mM SDS, pH 9.0, 10 mM TM-γ-CD, 10 mM SBE-β-CD, 18 kV, 15 °C, 254 nm	[124]
CZE-UV	Sitafloxacin (chiral impurities: *R,R,S*-sitafloxacin, *S,S,R*-sitafloxacin, *R,S,R*-sitafloxacin)	bulk substance	Screening FrFD BGE concentration, BGE pH, γ-CD concentration, Cu^2+^ concentration, d-phenylalanine concentration, temperature, voltage Optimization FCCD BGE pH, γ-CD concentration, Cu^2+^ concentration, d-phenylalanine concentration/chiral resolution, migration time Robustness testing: PBD *R,S,R*-sitafloxacin enantiomeric impurity determination at 0.1% level	15 mM phosphate BGE, pH 4.5, 15 mM d-phenylalanine, 20 mM CuSO_4_, 20 mM γ-CD, 15 kV, 25 °C, 297 nm	[125]
CZE-UV	Lenalidomide (chiral impurity: *R*-lenalidomide)	bulk substance	Optimization FCCD BGE concentration, temperature, voltage/chiral resolution *R*-lenalidomide impurity determination at 0.1% level	30 mM phosphate BGE, pH 6.5, 30 mM SBE-β-CD, 12 kV, 10 °C, 210 nm	[78]
CZE-UV	*R*-lansoprazole (chiral impurity *S*-lansoprazole), *R*-rabeprazole (chiral impurity *S*-rabeprazole)	bulk substance, capsules	Screening FrFD BGE concentration, BGE pH, SBE-β-CD concentration, γ-CD concentration, temperature, voltage Optimization CCD SBE-β-CD concentration, voltage, temperature/chiral resolution, migration time Robustness testing: PBD *R*-lansoprazole and *S*-rabeprazole enantiomeric impurity determination at 0.15% level	Lansoprazole: 25 mM phosphate BGE, pH 7.0, 10 mM SBE-β-CD, 20 mM γ-CD, 20 kV, 17 °C, 210 nm Rabeprazole: 25 mM phosphate BGE, pH 7.0, 15 mM SBE-β-CD, 30 mM γ-CD, 20 kV, 18 °C, 210 nm	[126]
CZE-UV	*S*-citalopram (chiral impurity *R*-citalopram)	bulk substance, tablets	Screening FrFD BGE concentration, BGE pH, CM-β-CD concentration, temperature, voltage, injection pressure Optimization FCCD CM-β-CD concentration, temperature, voltage/chiral resolution, migration time Robustness testing: PBD *R*-citalopram enantiomeric impurity determination at 0.05% level	25 mM phosphate BGE, pH 7.0, 3 mM CM-β-CD, 15 kV, 17.5 °C, 230 nm	[127]
CZE-UV	Linagliptin (chiral impurity *S*-linagliptin)	bulk substance	Screening FrFD BGE concentration, BGE pH, CM-β-CD concentration, temperature, voltage, injection time/chiral resolution, resolution between the internal standard and chiral impurity, migration time, peak height, distance between linagliptin peak and EOF Optimization I-Optimal design BGE concentration, BGE pH, CM-β-CD concentration, temperature, voltage/chiral resolution, migration time, peak height, distance between linagliptin peak and EOF Robustness testing: PBD *S*-linagliptin enantiomeric impurity determination at 0.05% level	70 mM sodium acetate BGE, pH 6.10, 4.7 mM CM-β-CD, 28 kV, 25 °C, 200 nm	[128]
CZE-UV	*R*-solriamfetol (chiral impurities: *S*-solriamfetol, *R*-phenylalaninol, *S*-phenylalaninol)	bulk substance, tablets	Screening FrFD BGE concentration, BGE pH, S-γ-CD concentration, temperature, voltage/chiral resolution, migration time, resolution between phenylalaninol enantiomers, resolution between *S*-solriamfetol enantiomeric impurity and *R*-phenylalaninol *S*-solriamfetol enantiomeric impurity determination at 0.1% level	45 mM Tris-acetate BGE, pH 4.5, 4 mM S-γ-CD, 19.5 kV, 21 °C, 200 nm	[79]

CZE-UV—capillary zone electrophoresis with UV detection; CZE–LIF—capillary zone electrophoresis with laser induced fluorescence detection; MEKC-UV—micellar electrokinetic capillary chromatography with UV detection. CCD—central composite design; FCCD—face-centered central composite design; FFD—full factorial design; FrFD—fractional factorial design, OD—orthogonal design, PBD—Plackett–Burman design. CM-β-CD—carboxymethyl-β-cyclodextrin; HP-β-CD—hydroxypropyl-β-cyclodextrin; S-γ-CD—sulfated-γ-CD; SBE-β-CD—sulfobutylether-β-CD; TM-γ-CD—heptakis(2,3,6-tri-O-methyl)-γ-cyclodextrin. DMSO—dimethyl sulfoxide, SDS—sodium dodecyl sulphate.

**Table 2:** Chiral CE methods for the chiral purity analysis of pharmaceuticals developed using AQbD approaches (2012–2022).

CE Technique	Pharmaceuticals	Matrix	DoE Methodology Factors/Responses	Design Space Determination/Validation/Optimized Analytical Conditions	References
CZE-UV	Levosulpiride (chiral impurity dextrosulpiride)	bulk substance, injection solutions	Asymmetric screening matrix BGE concentration, BGE pH, neutral CD concentration, type of neutral CD, S-β-CD concentration, voltage Optimization DD BGE pH, M-β-CD concentration, S-β-CD concentration, voltage/chiral resolution, migration time	Monte Carlo simulation Robustness testing: PBD *R*-sulpiride enantiomeric impurity determination at 0.1% level 5 mM Britton-Robinson BGE, pH 3.45, 10 mM S-β-CD, 34 mM M-β-CD, −14 kV, 16 °C, 214 nm	[129]
CZE-UV	*S*-Ambrisentan (chiral impurity *R*-ambrisentan)	bulk substance, real sample	Screening FrFD BGE concentration, BGE pH, γ-CD concentration, temperature, voltage Optimization FCCD BGE concentration, temperature, voltage/chiral resolution, migration time	Monte Carlo simulation Robustness testing: PBD *R*-ambrisentan enantiomeric impurity determination at 0.1% level 50 mM sodium acetate BGE, pH 4.0, 30 mM γ-CD, 25 kV, 25 °C, 200 nm	[130]
MEKC-UV	*S*-Ambrisentan (chiral impurity *R*-ambrisentan and three achiral impurities)	bulk substance, coated tablets	Asymmetric screening matrix BGE concentration, BGE pH, γ-CD concentration, SDS concentration, temperature, voltage, capillary length Optimization FCCD BGE pH, γ-CD concentration, voltage/chiral resolution, migration time	Monte Carlo simulation Robustness testing: PBD *R*-ambrisentan enantiomeric impurity determination at 0.1% level 100 mM borate BGE, pH 9.20, 100 mM SDS, 50 mM γ-CD, 30 kV, 22 °C, 200 nm	[131]
CZE-UV	Levomepromazine (chiral impurity dextromepromazine, levomepromazine sulphoxide diastereomers)	bulk substance, injection solution	Screening FrFD BGE concentration, BGE pH, HP-γ-CD concentration, temperature, voltage Optimization FCCD BGE concentration, BGE pH, HP-γ-CD concentration/chiral resolution expressed as selectivity, resolution, migration time	Monte Carlo simulation Robustness testing: PBD Dextromepromazine enantiomeric impurity determination in the 0.1–1.0% range (0.25 mg/mL levomepromazine) 100 mM citric acid BGE, pH 2.85, 3.6 mg/mL HP-γ-CD, 25 kV, 15 °C, 253 nm	[132]
CZE-UV	Dextromethorphan (chiral impurity levomethorphan)	bulk substance, capsules	Screening FrFD BGE concentration, BGE pH, M-α-CD concentration, S-β-CD concentration, temperature, voltage Optimization FCCDM-α-CD concentration, S-β-CD concentration, voltage/migration time, number of theoretical plates, height of levomethorphan, tailing factor of dextromethorphan	Monte Carlo simulation Robustness testing: PBD Levomethorphan enantiomeric impurity determination at 0.1% level 30 mM phosphate BGE, pH 6.5, 16 mg/mL S-β-CD, 14 mg/mL M-α-CD, 20 kV, 20 °C, 200 nm	[133]
CZE-UV (derivatization with dansyl chloride)	Pregabalin (chiral impurity *R*-pregabalin)	bulk substance, capsules	Screening D-optimal design BGE concentration, BGE pH, TM-β-CD concentration, temperature, voltage Optimization FCCD TM-β-CD concentration, temperature, voltage/chiral resolution, migration time	Monte Carlo simulation Robustness testing: PBD *R*-pregabalin enantiomeric impurity determination at 0.05% level 100 mM phosphate BGE, pH 2.5, 40 mg/mL TM-β-CD, 15 kV, 25 °C, 220 nm	[134]
CZE-UV	Dexmedetomidine (chiral impurity levomedetomidine)	bulk substance, tablets	Screening FrFD BGE concentration, BGE pH, S-β-CD concentration, temperature, voltage Optimization FCCD S-β-CD concentration, temperature, voltage/chiral resolution expressed as selectivity, migration time, current	Monte Carlo simulation Robustness testing: PBD Levomedetomidine enantiomeric impurity determination at 0.1% level 50 mM phosphate BGE, pH 6.5, 40 mg/mL S-β-CD, 10 kV, 17 °C, 200 nm	[135]
CZE-UV	Cinacalcet (chiral impurity *S*-cinacalcet and two chiral impurities)	bulk substance, tablets	Optimization BBD BGE pH, HP-γ-CD concentration, methanol concentration, voltage/chiral resolution, migration time	Monte Carlo simulation Robustness testing: PBD *S*-cinacalcet enantiomeric impurity determination at 0.1% level 150 mM phosphate BGE, pH 2.7, 3.1 mM HP-γ-CD, 2% (*v/v*) methanol, 26 kV, 18 °C, 220 nm	[136]
CZE-UV	*S*-dapoxetine (chiral impurities: *R*-dapoxetine, (*S*)-3-amino-3-phenylpropan-1-ol, (3*S*)-3-(dimethylamino)-3-phenylpropan-1-ol)	bulk substance, tablets	Screening FrFD BGE concentration, BGE pH, CD concentration (DM-β-CD: S-γ-CD 1:1), temperature, voltage Optimization FCCD DM-β-CD concentration, S-γ-CD concentration, voltage/chiral resolution, migration time, peak symmetry, current	Monte Carlo simulation Robustness testing: PBD *R*-dapoxetine enantiomeric impurity determination in the 0.05–1.0% range 50 mM phosphate BGE, pH 6.3, 40.2 mg/mL DM-β-CD, 45 mg/mL S-γ-CD, 9 kV, 15 °C, 215 nm	[137]
CZE-UV	Levodropropizine (chiral impurity dextrodropropizine and achiral precursor impurity)	bulk substance, pharmaceutical drops	Screening FFD S-β-CD concentration, 2-propanol concentration, temperature, voltage Optimization FCCD S-β-CD concentration, temperature/separation factors, migration time, current	Monte Carlo simulation Robustness testing: PBD dextrodropropizine enantiomeric impurity determination at 0.5% level 25 mM phosphate BGE, pH 7.0, 23.5 mg/mL S-β-CD, 10% (*v/v*) 2-propanol, 16.5 kV, 16.3 °C, 200 nm	[138]
NACE-UV	Levomepromazine (chiral impurity dextromepromazine)	bulk substance, tablets	Screening FrFD ammonium acetate concentration, acetic acid concentration, HDMS-β-CD concentration, temperature, voltage/separation factor, migration time, current Optimization FCCD ammonium acetate concentration, HDMS-β-CD concentration, voltage/chiral resolution, migration time, separation factors	Monte Carlo simulation Robustness testing: PBD dextromepromazine enantiomeric impurity determination in the 0.01–3.0% range (0.74 mg/mL levomepromazine) 75 mM acetic acid, 55 mM ammonium acetate in methanol BGE, 27.5 mg/mL HDMS-β-CD, 22 kV, 15 °C, 250 nm	[139]
CZE-UV	Tenofovir (chiral impurity *S*-tenofovir)	bulk substance	Screening FrFD BGE concentration, BGE pH, QA-β-CD concentration, temperature, voltage Optimization FCCD BGE pH, temperature, voltage/migration time, separation factor, current	Monte Carlo simulation Robustness testing: PBD *S*-tenofovir enantiomeric impurity determination at 0.1% level 100 mM phosphate BGE, pH 6.4, 45 mg/mL QA-β-CD, 18 kV, 22 °C, 257 nm	[140]

CZE-UV—capillary zone electrophoresis with UV detection; MEKC-UV—micellar electrokinetic capillary chromatography with UV detection; NACE-UV—non-aqueous capillary electrophoresis with UV detection. BBD—Box–Behnken design; DD—Doehlert design; FCCD—face-centered central composite design; FFD—full factorial design; FrFD—fractional factorial design, PBD—Plackett–Burman design. DM-β-CD—dimethyl-β-cyclodextrin; HDMS-β-CD—heptakis-(2,3-di-O-methyl-6-O-sulfo)-β-cyclodextrin; HP-γ-CD—hydroxypropyl-γ-cyclodextrin; M-α-CD—methyl-α-CD; M-β-CD—methyl-β-CD; QA-β-CD—quaternary ammonium β-CD; S-β-CD—sulfated-β-CD; S-γ-CD—sulfated-γ-CD; SDS—sodium dodecyl sulfate; TM-β-CD—heptakis(2,3,6-tri-O-methyl)-β-cyclodextrin.

## Data Availability

Not applicable.

## Sample Availability

No new samples were generated for this review.

## Abbreviations

ACE—affinity capillary electrophoresis; ANOVA—Analysis Of Variance; AP—analytical procedure; API—Active Pharmaceutical Ingredient; AQbD—Analytical Quality by Design; ATP—Analytical Target Profile; BBD—Box–Behnken design; BGE—background electrolyte; CCD—central composite design; CD—cyclodextrin; CD-MEEKC—cyclodextrin-modified microemulsion electrokinetic chromatography; CD-MEKC—cyclodextrin-modified micellar electrokinetic chromatography; CE—capillary electrophoresis; CM-β-CD—carboxymethyl-β-cyclodextrin; CMA—Critical Method Attribute; CMP—Critical Method Parameter; CS—chiral selector; CZE– capillary zone electrophoresis; DD—Doehlert design; DL—Detection Limit; DM-β-CD—dimethyl-β-cyclodextrin; DMSO—dimethyl sulfoxide; DoE—Design of Experiments; DP—drug product; DS—Design Space; EC—Established Conditions; EKC—electrokinetic chromatography; EMO—enantiomer migration order; EOF—electroosmotic flow; FCCD—face-centered central composite design; FDA—Food and Drug Administration; FFD—full factorial design; FLEC—1-(9-fluorenyl)ethyl chloroformate; FMEA—Failure Mode and Effect Analysis; FrFD—fractional factorial design; GC—gas chromatography; HDMS-β-CD—heptakis-(2,3-di-O-methyl-6-O-sulfo)-β-cyclodextrin; HP-β-CD—hydroxypropyl-β-cyclodextrin; HP-γ-CD—hydroxypropyl-γ-cyclodextrin; ICH—International Council for Harmonisation; IL—ionic liquid; LC—liquid chromatography; M-α-CD—methyl-α-CD; M-β-CD—methyl-β-CD; MEKC—micellar electrokinetic capillary chromatography; MODR—Method Operable Design Region; NACE—non-aqueous capillary electrophoresis; NMR—nuclear magnetic resonance; OFAT—One Factor At a Time; OD—orthogonal design; QA—quality assurance; QA-β-CD—quaternary ammonium β-CD; QbD—Quality by Design; QbT—Quality by Testing; QC—quality control; QL—Quantitation Limit; QRM—Quality Risk Management; PAR—Proven Acceptable Ranges; PBD—Plackett–Burman design; PC—performance characteristic; RPN—Risk Priority Number; RR—Reportable Result; RSM—Response Surface Methodology; S-β-CD—sulfated-β-CD; S-γ-CD—sulfated-γ-CD; SBE-β-CD—sulfobutylether-β-CD; SDS—sodium dodecyl sulfate; SFC—supercritical fluid chromatography; SST—system suitability test; TM-β-CD—heptakis(2,3,6-tri-O-methyl)-β-cyclodextrin; TM-γ-CD—heptakis(2,3,6-tri-O-methyl)-γ-cyclodextrin.
